# Invasive fall armyworms are corn strain

**DOI:** 10.1038/s41598-024-56301-0

**Published:** 2024-03-08

**Authors:** Karine Durand, Hyerin An, Kiwoong Nam

**Affiliations:** DGIMI, Univ Montpellier, INRAE, Montpellier, France

**Keywords:** Corn strain, Fall armyworm, Invasion, Pest genomics, *Spodoptera frugiperda*, Evolutionary genetics, Agroecology, Invasive species

## Abstract

The fall armyworm (*Spodoptera frugiperda*) is one of the major pest insects in diverse crop plants, including maize, rice, and cotton. While the fall armyworm is native to North and South America, its invasion was first reported in West Africa in 2016. Since then, this species has rapidly spread across Sub-Saharan Africa, Asia, and Oceania, as well as Egypt and Cyprus. The fall armyworm is composed of two sympatric strains, the corn and rice strains, designated to their preferred host plants, in native areas. It remains surprisingly unclear whether invasive fall armyworms belong to the corn strain, rice strain, or hybrids of the two, despite a large number of population genetics studies. In this study, we performed population genomics analyses using globally collected 116 samples to identify the strains of invasive fall armyworms. We observed that invasive fall armyworms are genomically most similar to the corn strain. The reconstructed phylogenetic tree supports the hypothesis that invasive fall armyworms originated from the corn strain. All genomic loci of invasive populations exhibit higher genetic similarity to the corn strains compared to the rice strains. Furthermore, we found no evidence of gene flow from rice strains to invasive populations at any genomic locus. These results demonstrate that invasive fall armyworms belong to the corn strain. These results suggest that invasive fall armyworms likely have very limited potential to infest rice. Therefore, the management plan should primarily focus on crops preferred by the corn strain.

## Introduction

The fall armyworm (*Spodoptera frugiperda*; Noctuidea; Lepidoptera, FAW) is one of the major pest insects of diverse crops including cotton, maize, rice, and sorghum. FAW is native to North and South America, and its invasion was first reported in West Africa in 2016^1^. Since then, FAW has rapidly spread to various regions, including Sub-Saharan Africa, South Asia, Southeast Asia, East Asia, Oceania, Egypt, and more recently, Cyprus and the Canary Islands (https://www.fao.org/fall-armyworm/monitoring-tools/faw-map/en/). Invasive FAWs cause significant yield losses in maize production. For example, FAW reduced up to 23–53% of maize production in Sub-Saharan Africa^2^, where maize provides at least 30% of total caloric intake^3^. Field-evolved resistance to insecticides appears to be widespread^4–9^, posing challenges for FAW control. Given the global and urgent nature of the FAW invasion, it is crucial to monitor and assess the negative impacts of invasive FAWs, as well as to develop sustainable and ecologically sound methods for FAW control.

While FAWs exhibit extreme polyphagy by eating more than 353 plants in 76 families^10^, FAW consists of two strains with distinct ranges of host plants^11,12^. The corn strain (sfC) has a preference for corn, cotton, and sorghum, while the rice strain (sfR) prefers alfalfa, millet, pasture grasses, and rice^13^. Both sfC and sfR are sympatrically observed throughout almost the entire native habitat range. Reciprocal transplant experiments have demonstrated differential adaptation to host plants between sfC and sfR^14^. sfC and sfR showed a clear pattern of genetic differentiation across the whole genome sequences, indicating the presence of incipient speciation driven by differential host plant adaptation^15,16^. Since sfC and sfR are morphologically indistinguishable, molecular markers have been utilized to identify the strains. These molecular markers include the Z chromosome *triosephosphate isomerase* (TPI) gene^17^ and the mitochondrial *cytochrome c oxidase subunit 1* (COX1) gene^18^.

The taxonomic identification of invasive FAWs is one of the most basic pieces of information to monitor invasive FAWs and assess their negative impacts. However, it is surprisingly unclear to which strains invasive FAWs belong, even though a large number of related studies have been performed. Previous population genetics studies suggested that invasive FAW populations are hybrids between the sfC and sfR strains based on the presence of sfC-type Z chromosome TPI and sfR-type mt-COX1 genes in invasive samples^19–22^. Population genetics studies based only on mt-COX1 genes concluded that sfR invaded^23,24^. However, with this conclusion, it is hard to explain why invasive FAWs are predominantly detected from sfC-preferred crops^25^, such as maize. Furthermore, it remains unclear whether invasive FAWs have the potential to cause a reduction in rice production, even when a serious infestation of rice fields has never been reported. This ambiguity may be attributed to the limited discriminative capacity of the mt-COX1 or TPI genes in identifying strains. Our whole genome analyses have revealed that, while the TPI gene can reliably distinguish between strains, the mt-COX1 gene can only differentiate between two sub-strains within sfC^16^. Therefore, it is imperative to reevaluate the widespread belief that invasive FAWs are hybrids or sfRs through whole genome analyses.

**Figure 1:**
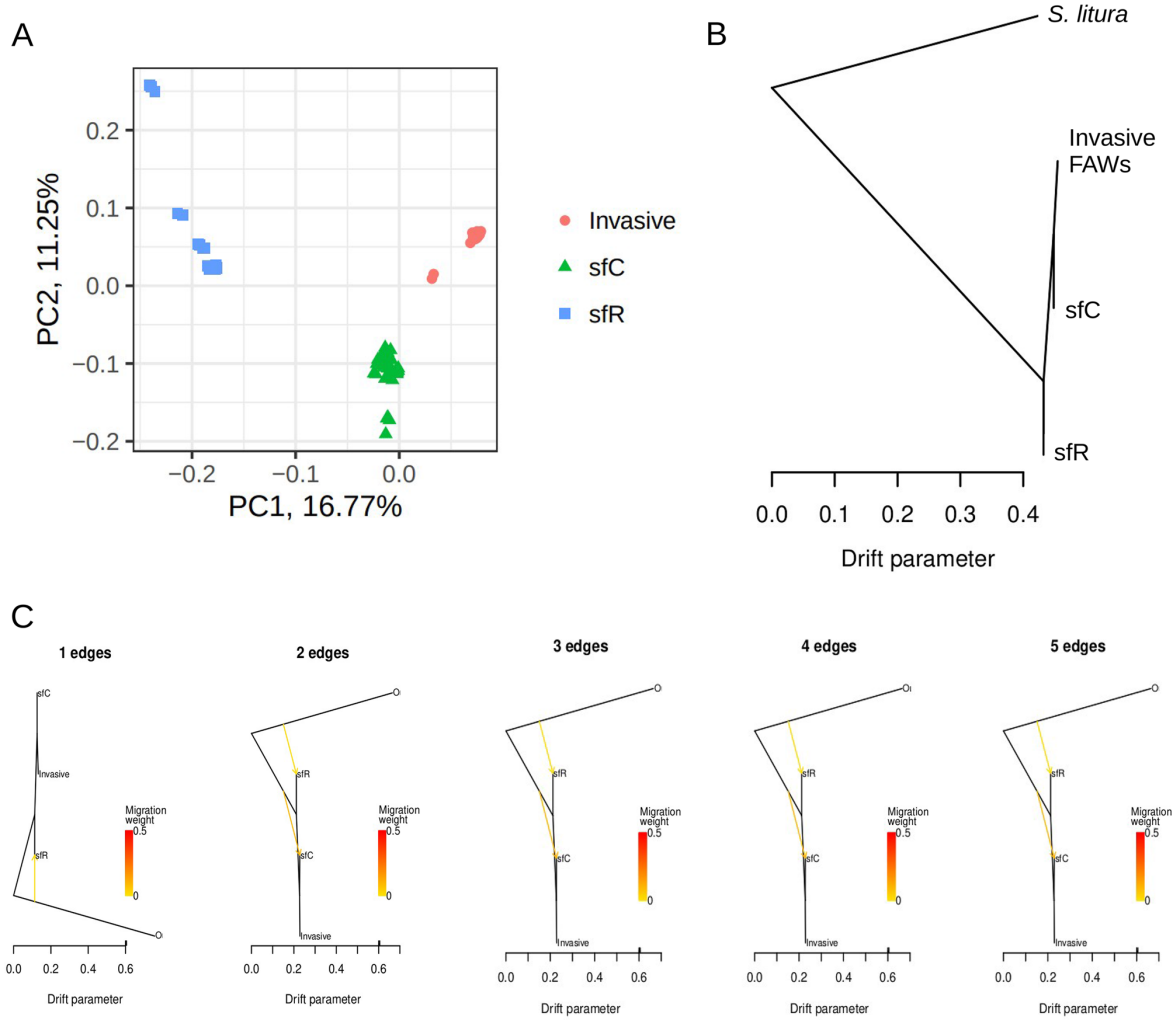
The phylogeny of FAW populations. (**A**) Principal component analysis revealed clear groupings of FAW individuals into three groups: sfC, sfR, and invasive populations. This figure was regenerated with the modification of Yainna et al.^26^ to visualize the population structure of FAW. A clear grouping among sfC, sfR, and invasive populations is shown. Along the first principal component, sfC appeared closer to invasive populations than sfR. (**B**) The TreeMIX analysis indicated that ancestral FAWs split into sfR and sfC + invasive FAWs, suggesting that invasive FAWs originated from sfC. (**C**) Gene flow from sfR to invasive populations was not detected with a range of edge numbers.

We conducted whole-genome sequencing analysis on 177 samples collected globally, aiming to infer the evolutionary history of FAW invasions^26^. We showed clear genomic differentiation between the sfC and sfR strains in native FAW populations. We also observed that invasive populations are genomically closest to native sfC samples. We conclude that invasive populations should be considered as sfC strains rather than hybrids (Fig. 1A). This conclusion is indeed in line with the observation that invasive FAWs are observed almost exclusively from host plants preferred by sfC^25^. Since the observed genomic similarity reflects the averaged pattern across the whole genomes, it remains still possible that a part of invasive genomes originated from sfR through ancient gene flow while the majority of genomic sequences have sfC-type sequences. In such a scenario, invasive FAWs could be considered to be partial hybrids. This study aims to determine the strain of invasive populations by testing the existence of sfR-type genomic loci in invasive populations using publicly available whole genome sequences generated by our previous study. If the invasive FAW genome contains a part of sfR-derived sequences, infestation to rice could be considered for monitoring. However, if such a genetic introgression from sfR to the invasive populations is not observed, the risk of infesting rice should be considered to be low, as native sfCs^16^.

## Results

A phylogenetic tree inferred using TreeMix analysis showed that FAWs were divided into sfC + invasive populations and sfR (Fig. 1B). Together with Fig. 1A, these results showed that invasive populations are genetically closer to sfC than sfR, and invasive FAWs originated from sfC as shown by Yainna et al.^26^. According to the TreeMix analyses, gene flow from sfR to invasive populations was not detected in a range of edge numbers (Fig. 1C). The *f3* statistic (sfC, sfR; invasive FAWs) was significantly higher than zero (0.038494; z-score = 32.544; p-value < 0.001), indicating that invasive FAWs are not genetically admixed between sfC and sfR.

**Figure 2:**
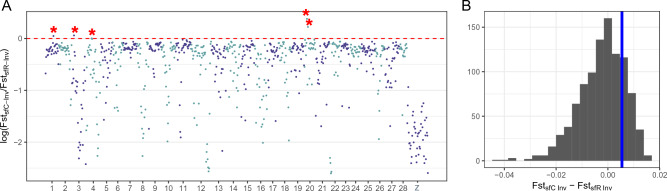
Invasive populations are genetically closer to sfC than sfR across the whole genome. (**A**) The log-transformed ratio of F_ST_ between sfC and invasive populations (Fst_sfC-inv_) to F_ST_ between sfR and the invasive population (Fst_sfR-inv_) is represented. If Fst_sfC-inv_ < Fst_sfR-inv_, the log-transformed ratio will be below zero, indicated by the horizontal red dotted bar. Five 500 kb windows with Fst_sfC-inv_ > Fst_sfR-inv_ are marked with red asterisks. (**B**) The vertical blue bar represents the average difference between Fst_sfC-inv_ and Fst_sfR-inv_ across the five windows. The histogram illustrates the difference of F_ST_ calculated from two randomly generated native groups. The proportion of random groups with higher values than Fst_sfC-inv_ − Fst_sfR-inv_ corresponds to the p-values.

Then, we tested the presence of invasive genomic loci that are more similar to sfR than sfC. The genomic average F_ST_ between invasive FAWs and sfR was 0.115, which was higher than the F_ST_ between invasive FAWs and sfC (0.0426). F_ST_ was also calculated from non-overlapping 500 kb windows across the genome to identify loci at which invasive populations have higher F_ST_ with sfC than with sfR. Among a total of 782 non-overlapping 500 kb windows across the genome (Fig. 2), only five windows showed such a pattern, corresponding to 0.639% of the whole genome sequences. The average F_ST_ across these five windows was 0.0532 and 0.0478 between sfC and invasive populations and between sfR and invasive populations, respectively. However, statistical differences between these two F_ST_ values were not supported (p-value = 0.249; one-sided randomization test with 1,000 replications). This result implies that no genomic locus in invasive populations has more similar sequences to sfR than sfC with statistical significance.

**Figure 3:**
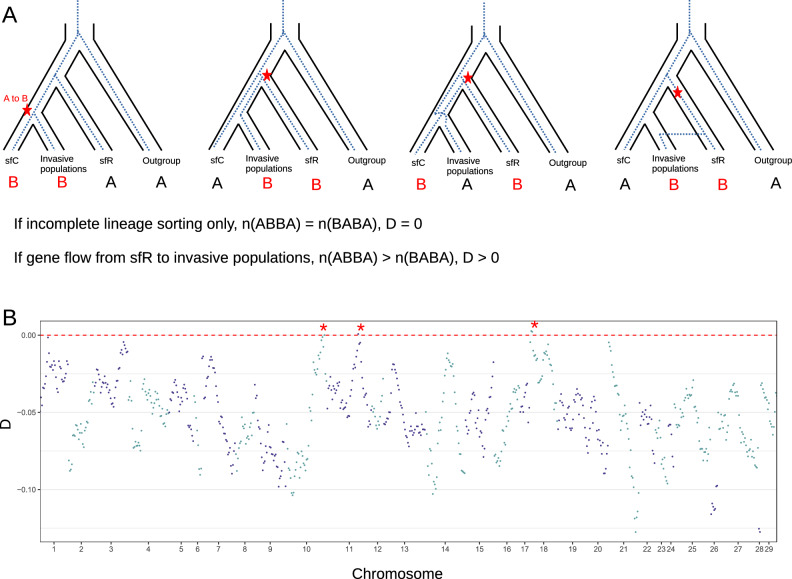
Testing gene flow from sfR to invasive populations. (**A**) If a mutation that changes from A to B originated from the common ancestor between sfC and invasive populations (indicated by the red stars), the genotype combination among sfC, invasive populations, sfR, and the outgroup will be BBAA. In the presence of incomplete lineage sorting, the gene tree may differ from the FAW strain tree in Fig. 1B. In such cases, when the frequency of ABBA and BABA will be the same. Then, D statistics, normalized differences between the frequency of ABBA and BABA will be zero. In the presence of gene flow from sfR to invasive populations, the invasive populations will acquire mutations generated in the sfR genealogy. As a result, the frequency of ABBA will be higher than that of BABA. Consequently, the D statistic will be higher than zero. (**B**) The majority of D statistics calculated across the genome are below zero, indicating a lack of gene flow. However, there are three loci with slightly higher D statistics than zero, as denoted by the red asterisks.

We also tested the existence of loci with genetic footprints of gene flow from sfR to the invasive population using D statistics. If incomplete lineage sorting caused an incongruent pattern between the phylogenetic tree shown in Fig. 1B and the gene tree inferred from the distribution of alleles, then, the same frequency of genetic variations will be observed between ABBA and BABA patterns, generating D statistics equal to zero^27^ (Fig. 3A). Alternatively, if gene flows from sfR to invasive population existed, then, the ABBA pattern will outnumber the BABA pattern, making D statistics higher than zero. D statistics calculated from the whole genome sequences had a negative sign (-0.0540), implying that gene flow was not detected globally.

When D statistics were calculated from 10,000 SNV windows, only three loci in three chromosomes had D statistics higher than zero (Fig. 3B). These three loci windows correspond to 0.539% of all analyzed loci in the genomes. These loci do not have D statistics higher than zero with statistical significance (Z-test, p = 0.7225).

## Discussion

In this study, we aim to perform unambiguous taxonomic identification of invasive FAWs among sfC, sfR, and their hybrids using whole genome sequences from globally sampled 116 individuals. Phylogenetic analysis showed that invasive FAWs originated from sfC (Fig. 1), as shown by Yainna et al.^26^. F_ST_ statistics showed that invasive FAWs have no genomic locus that is closer to sfR than sfC (Fig. 2). According to the D statistics, gene flow from sfR to the invasive populations was not supported by any genomic locus (Fig. 3). Taken together, these results demonstrate that invasive FAWs correspond to sfC without genetic introgression from sfR. Therefore, we argue that invasive FAWs probably have a very limited capacity to infest rice, while this risk has been of great interest in invaded area^28,29^.

We argue that the misinterpretation of sfR-type mt-COX1 genes may have led to the conclusion that invasive FAWs are hybrids. This conclusion was based on the observation that the majority of invasive FAWs exhibit the sfC type according to the TPI marker and the sfR type according to the mt-COX1 genes^19–22^. Fiteni et al. showed through population genomics analyses conducted on FAW larvae collected from different host plants that FAW can be divided into two strains: sfC and sfR. Additionally, within the sfC subgroup, two sub-strains, mtA and mtB, have been identified, each with distinct mitochondrial and nuclear genomes^16^. Furthermore, they  demonstrated that mitochondrial sequences are indistinguishable between mtB and sfR, whereas mtA and mtB exhibit clear differentiation in mitochondrial sequences. Therefore, the presence of sfR-type mt-COX1 genes in invasive FAWs may correspond to the mtB sub-strain, rather than sfR. Taking into consideration the absence of gene flow from sfR to invasive populations, as observed in this study, we argue that this possibility is likely true. Thus, we also raise the possibility that population genetics studies, by relying solely on mt-COX1 genes, might have been misled into concluding that sfR were invading^23,24^.

One possible criticism against our conclusion is the potential occurrence of gene flow from sfR to invasive populations that were not included in this study. In this case, a proportion of invasive populations might be hybrids. Zhang et al.^30^. performed population genomics analysis using an independent resequencing dataset consisting of 280 FAW samples collected from five native areas (the mainland USA, Brazil, Guadeloupe, Argentina, and Puerto Rico) and eight invasive areas (China, Ethiopia, Ghana, Kenya, Malawi, Rwanda, South Africa, and Zambia). They reported that invasive populations are genetically closest to sfC while also reporting genomic differentiation among invasive populations, as shown by Yainna et al. The consistently observed patterns from different resequencing datasets covering wide geographical ranges suggest that a ghost population of sfR is unlikely to exist in the invaded area. Therefore, the inclusion of additional invasive populations is unlikely to change our conclusion in this study, even though we cannot absolutely exclude the possibility of ancient gene flow from sfR to unknown invasive populations.

In this study, we showed that invasive FAWs are pure sfC without gene flow from sfR. This result implies that invasive FAWs are not likely to cause massive infestation on sfR-preferred plants, such as rice or grasses. Even though invasive FAWs may be anecdotally observed from rice, we do not believe that invasive FAWs will establish permanent populations on these plants without a special evolutionary force expanding the range of host plants. This argument does not imply that it is unnecessary to perform surveillance on sfR from invaded areas because sfR might arrive at invaded areas through additional introductions^22^. Instead, our study indicates that we need to consider the limited capacity of FAWs to infest rice when developing FAW monitoring or prevention plans.

## Methods

This study is based on the whole genome resequencing data generated by Yainna et al.^26^. The dataset consisted of 177 samples collected from 12 geographic locations, including Brazil, Florida, French Guinea, Guadeloupe, Mexico, Mississippi, and Puerto Rico for native areas and Benin, China, India, Malawi, and Uganda for invaded areas. The accession numbers to the raw reads were PRJNA494340, PRJNA577869, PRJNA639295, and PRJNA639296 in NCBI SRA. Please see Yainna et al. for detailed information. We excluded samples from Brazil, Malawi, and Uganda because the data was not publically available when we performed the analyses^8,31^. Mexican samples were also excluded as they were phylogenetically distinct from the others. Additionally, one hybrid sample from Florida (FGJ4) and one sfR sample from a corn field in Mississippi (MS_R_R6) were excluded to avoid interference from local hybridization or migration between crop fields. The total number of analyzed samples was 116. These samples include 44 native sfC ones from Florida (13 samples), Mississippi (16), and Puerto Rico (15), 17 native sfR ones from Florida (10), French Guiana (3), and Guadeloupe (4), and 55 invasive ones from Benin (39), China (2), and India (14). The resequencing dataset includes 27,117,672 single nucleotide variations (SNVs) across whole-genome sequences from these 116 samples.

Principal component analysis was conducted using Plink v1.9^32^. A phylogenetic tree among sfC, sfR, and invasive populations was inferred using TreeMIX v1.13^33^. Whole genome sequences of *S. litura* were used as an outgroup (NCBI Accession: SRR5132437). F_ST_^34^ was calculated between sfC and invasive populations and between sfR and invasive populations from non-overlapping 500 kb windows using VCFtools v0.1.15^35^. The *f3* statistic^27^ was calculated to test the genetic admixture of invasive FAWs between sfC and sfR, using the following method. Initially, SNVs in the vcf file were excluded if the genotype was undetermined in any sample or if the SNV was non-biallelic using VCFtools v0.1.15^35^. Subsequently, the filtered VCF file was converted to PLINK format using VCFtools v0.1.15, followed by additional conversion to eigenstrat format using CONVERTF in the admixtools package v7.02^27^. Finally, the *f3* statistic and the corresponding z-score were calculated using qp3Pop the admixtools package v7.02 in conjunction with admixr v0.9.1^36^. The p-value was derived from the Z table. The number of used SNVs was 232,518 in this analysis. D statistics indicating gene flow from sfR to invasive populations were computed from sliding windows using Dsuit v0.4 r38 with Dinvestigate option^37^. The number of SNVs was 10,000 for each window, and the step size was 1,000 SNVs. The statistical significance of D statistics greater than 0 was tested using Comp-D v6695c6b^38^ from Z-statistics.

## Data Availability

All data generated or analyzed during this study are included in this published article^26^. The NCBI SRA accession numbers of this project are PRJNA494340, PRJNA577869, PRJNA639295, and PRJNA639296. The computer programming scripts used during the current study are available from the corresponding author on request.
